# Comparison of solvate ionic liquids and DMSO as an *in vivo* delivery and storage media for small molecular therapeutics

**DOI:** 10.1186/s12896-018-0442-1

**Published:** 2018-05-29

**Authors:** Prusothman Yoganantharajah, Alexander P. Ray, Daniel J. Eyckens, Luke C. Henderson, Yann Gibert

**Affiliations:** 1 Metabolic Genetic Diseases Laboratory, Metabolic Research Unit, Deakin University School of Medicine, 75 Pigdons Road, Geelong, VIC 3216 Australia; 2 Institute for Frontier Materials, Deakin University, 75 Pigdons Road, Geelong, VIC 3216 Australia

**Keywords:** Zebrafish, Embryogenesis, Retinoic acid, Aldh1a2, FGFR, DEAB, SU5402, Ionic liquids

## Abstract

**Background:**

Solvate ionic liquids (SILs) are a new class of ionic liquids that are equimolar solutions of lithium bistrifluoromethanesulfonimide in either triglyme or tetraglyme, referred to as G3LiTFSA and G4LiTFSA, respectively. SILs play a role in energy storage lithium batteries, and have been proposed as potential alternatives to traditional organic solvents such as DMSO. G3TFSA and G4TFSA have been shown to exhibit no toxicity *in vivo* up to 0.5% (v/v), and solubilize small compounds (N,N-diethylaminobenzaldehyde) with full penetrance, similar to DMSO delivered DEAB. Herein, we compare the effects of storage (either at room temperature or − 20 °C) on DEAB solubilized in either DMSO, G3TFSA or G4TFSA to investigate compound degradation and efficacy.

**Results:**

The findings show that DEAB stored at room temperature (RT) for 4 months solubilized in either G3TFSA, G4TFSA or DMSO displayed no loss of penetrance. The same was observed with stock solutions stored at − 20 °C for 4 months; however G4TFSA remained in a liquid state compared to both G3TFSA and DMSO. Moreover, we examined the ability of G3TFSA and G4TFSA to solubilize another small molecular therapeutic, the FGFR antagonist SU5402. G4TFSA, unlike G3TFSA solubilized SU5402 and displayed similar phenotypic characteristics and reduced dlx2a expression as reported and shown with SU5402 in DMSO; albeit more penetrative.

**Conclusion:**

This study validates the use of these ionic liquids as a potential replacement for DMSO *in vivo* as organic solubilizing agents.

## Background

In recent years, there has been an increase in the interest of ionic liquids (ILs) due to their potential in a myriad of chemical processes. Their unique property of being molten salts at room temperature imparts unusual properties such as; high ionic conductivity, non-flammability, and negligible vapour pressure. Due to these properties, and their high customisability through anion/cation pairing, have become a staple material used throughout a variety of disciplines [1–9]. The use of imidazolium ionic liquids in drug delivery has seen some success, as they offer the potential to deliver sparingly soluble molecules via oral, or transdermal routes over a long period of time [10–17]. Complementing this effect is the conversion of active pharmaceutical ingredients to ionic liquid-like salts, typically by inclusion of a charge diffuse cation or anion, which can result in improved therapeutic effect via changes in crystal structure [18, 19]. These approaches have largely revolved around the use of imidazolium-derived ionic liquids which are a well-used and studied class of solvents.

**Fig. 1:**
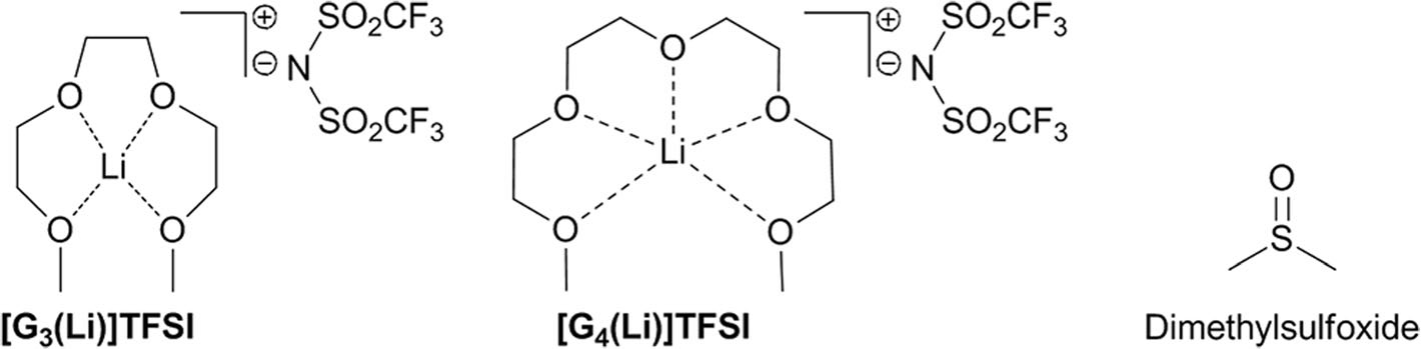
Solvate ionic liquids and DMSO which are the focus of this study

Of particular interest has been a new class of ionic liquids, termed ‘solvate ionic liquids’ (SILs), reported by Watanabe et al. [20–26]. The preparation of SILs is trivial, being simple dissolution of LiNTf_2_ (lithium bistrifluoromethanesulfonimide) in either triglyme (triethylene glycol dimethyl ether, G3) or tetraglyme (tetraethylene glycol dimethyl ether, G4) yields the ionic liquids, G3TFSA or G4TFSA, respectively (Fig. 1). Recent work by our group, and others, has characterised these solvate ionic liquids using Kamlet-Taft parameters, and explored their use as a solvent for organic chemical transformations [27, 28]. Recently, we demonstrated the toxicity of solvate ionic liquids *in vivo* using a zebrafish (*Danio rerio*), and found that both G3TFSA and G4TFSA with concentrations up to 50 μM (or 0.5%) are not toxic to zebrafish embryos (which are more sensitive to toxicity than adults) [29]. Since most organic modifier solvents (such as DMSO) are used at a much lower concentration (usually 0.1%), our study was able to conclude that both G3TFSA and G4TFSA can be safely used as aqueous modifier solvents to allow evaluation of small molecules. Both G3TFSA and G4TFSA do not induce apoptosis at a similar concentration to DMSO (10 μM) and display a full drug penetrance and the anticipated physiological changes induced in the test specimens [29]. Since these novel SILs were able to replace DMSO as organic modifiers, we were curious if they could be used as an alternative long term storage media for these molecules, and if compound degradation over this period was reduced compared to DMSO.

Currently, the most commonly used organic solvent, in academic and industrial research laboratories, to solubilize small organic molecules in water is DMSO [30]. Due to its ability to dissolve many kinds of compounds, DMSO plays a pivotal role in sample management and high-throughput screening during *in vivo* and *in vitro* evaluation.

Due to its broad solubilizing capability and apparent low toxicity at concentrations < 10%, [31, 32] DMSO is used as a solvent for many drug types and is used as the vehicle control of choice for both *in vitro* and *in vivo* studies. However, in a study coordinated by Corderio et al., the authors demonstrated low-dose toxicity of DMSO and concluded that solvents other than DMSO should be employed for solubilizing drugs [33]. Thus there is a need to find a suitable replacement for DMSO which do not possess this inherent toxicity.

Therefore, the focus and aim of this study was to evaluate the performance of the ILs G3TFSA and G4TFSA as a potential replacement to the conventional solvent DMSO, using zebrafish as a model organism. Since, we have shown the ability of G3TFSA and G4TFSA to solubilize DEAB with full penetrance of reported phenotypic characteristics [29]; we wanted to evaluate the impact of storage on both G3TFSA and G4TFSA, and their ability to solubilize and deliver other small molecules/pharmacological compounds.

## Results

### Evaluation of SILs stored at -20 °C

To be consistent with our previous study, which used the retinoic acid synthesis inhibitor 4-diethylaminobenzaldehyde (DEAB), we continued to use this molecule as the initial focus of this work. DEAB is a known inhibitor of retinaldehyde dehydrogenases ALDH1A1, ALDH1A2, and ALDH1A3 in mammals (teleost fish do not have an *aldh1a1* gene) [34]. Retinaldehyde dehydrogenases convert retinaldehyde, a product from retinol (Vitamin A1), into retinoic acid (RA) [35]. Hence, inhibiting the function of retinaldehyde dehydrogenases via DEAB abolishes the synthesis of RA required for normal growth and development [36]. Thus, we wanted to evaluate G3TFSA and G4TFSA’s ability to keep DEAB stable at RT without compound degradation or loss of efficacy. The rationale behind this test is that stock solutions kept at RT would remain in a liquid state with no concern for sample degradation from multiple freeze/thaw cycles. This was evaluated over a duration of 4 months, and differences in penetrance of the drug, measured by the strength of the phenotypes were determined.

To conduct this comparison, *N,N*-diethylaminobenzaldehyde (DEAB), was administered in parallel to zebrafish embryos: one sample containing DMSO only (0.1%), one DEAB in DMSO and the other two, containing DEAB in either G3TFSA or G4TFSA. Zebrafish embryos were exposed to DEAB in the respective solvents at the beginning of gastrulation occurring at 6 h post fertilization (hpf) at a final concentration of 5 μM.

**Fig. 2:**
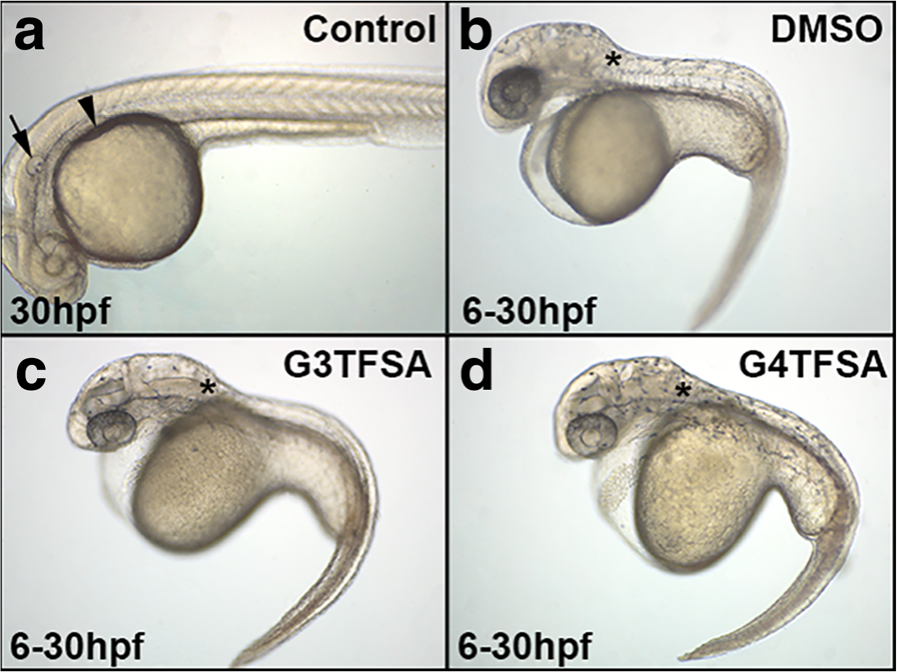
Room temperature DEAB exposure in developing zebrafish embryos. Embryos exposed to DEAB at 5 μM in solution at room temperature for 4 months in either DMSO (**b**), G3TFSA (**c**), or G4TFSA (**d**) displayed the reported characteristic of loss of RA (compared to control (DMSO exposed only (**a**): lack of pectoral fin induction (arrowhead), shortening of the posterior head (asterisk), malformation of the otic vesical (arrow) and pericardial edema

Comparison of the treatments kept at RT showed that zebrafish embryos exposed to DEAB solubilised in either DMSO (Fig. 2b), G3TFSA (Fig. 2c), or G4TFSA (Fig. 2d) from 6 to 30 hpf displayed characteristics associated with the loss of RA signalling compared to the control (Fig. 2a): [29, 37, 38] lack of pectoral fin induction (arrowhead), shortening of the posterior head (marked by an asterisk) malformation of the otic vesicle (arrow) and pericardiac oedema. There was no discernible differences in DEAB penetrance between DEAB in either DMSO, G3TFSA or G4TFSA (100% for each, *n* = 60) after storage at RT for 4 months [29].

### Evaluation of SILs stored at room temperature

**Fig. 3:**
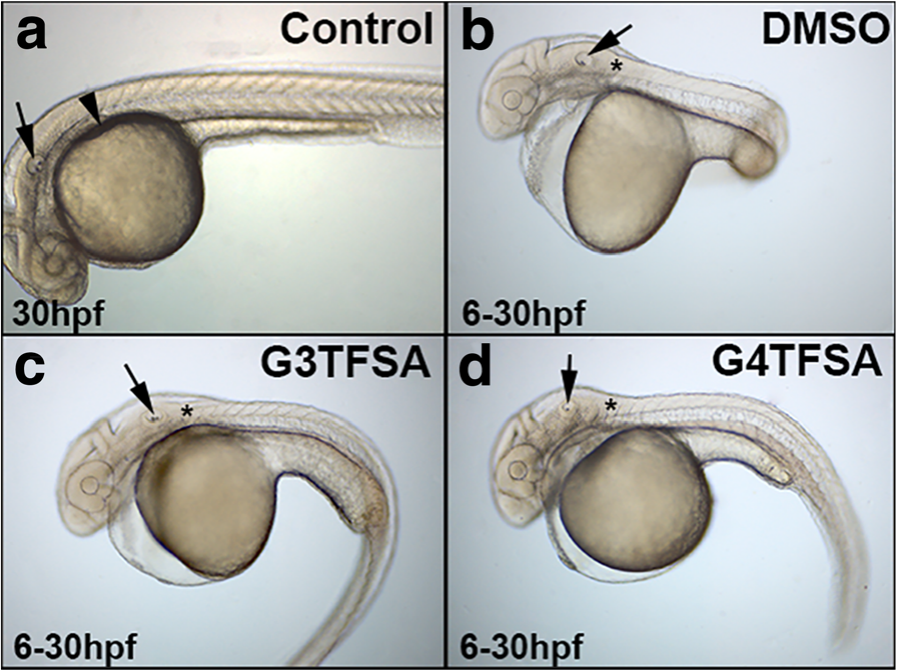
Frozen DEAB exposure in developing zebrafish embryos. Embryos exposed to DEAB at 5 μM that have been stored for 4 months at -20 °C in either DMSO (**b**), G3TFSA (**c**), or G4TFSA (**d**) display the reported characteristics of loss of RA (compared to control (DMSO exposed only (**a**): lack of pectoral fin induction (arrowhead), shortening of the posterior head (asterisk), malformation of the otic vesical (arrow) and pericardial edema

To assess the solvate properties of G3TFSA and G4TFSA, it was also imperative to evaluate the performance of the ionic liquids G3TFSA and G4TFSA after storage in the conventional (frozen) manner. Therefore, DEAB was stored at − 20 °C for 4 months in either DMSO, G3TFSA or G4TFSA. A comparison of zebrafish embryos treated with 5 μM showed that zebrafish embryos exposed to DEAB solubilised in either DMSO (Fig. 3b), G3TFSA (Fig. 3c), or G4TFSA (Fig. 3d) from 6 to 30 hpf displayed the characteristics associated with the loss of RA signalling compared to the control (Fig. 3a) [29, 37, 38] (100% penetrance for all compounds tested, *n* = 60).

As previously observed in the RT test, there was an observed phenotype that clearly showed a lack of pectoral fin induction (arrowhead), shortening of the posterior head (marked by an asterisk), malformation of the otic vesicle (arrow) and pericardiac oedema. However, unlike both DMSO and G3TFSA, G4TFSA did not freeze at − 20 °C but remained in a viscous state.

### Capacity of the SILs to deliver small compounds

**Fig. 4:**
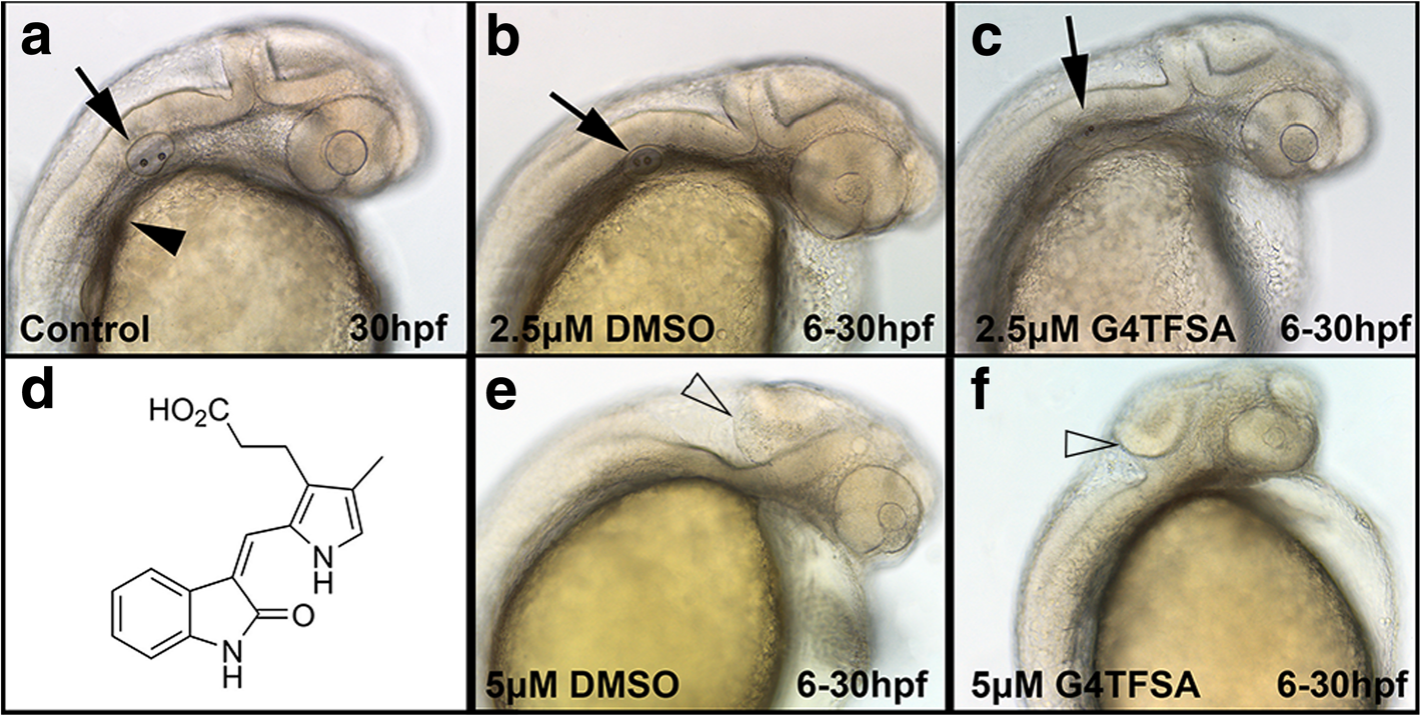
SU5402 exposure in developing zebrafish embryos. Embryos are exposed to SU5402 (**d**) at 2.5 μM (**b** & **c**) or 5 μM (**e** & **f**) in either DMSO or G4TFSA display the reported characteristics of FGF signaling inhibition compared to control (untreated (**a**) or DMSO exposed only (**b** & **e**)): lack of pectoral fin induction (marked by an asterisk), malformation of the otic vesicle (arrow). Lloss of MHB (open arrow head) was observed in embryos treated with 5 μM DEAB. 100× magnification

**Fig. 5:**
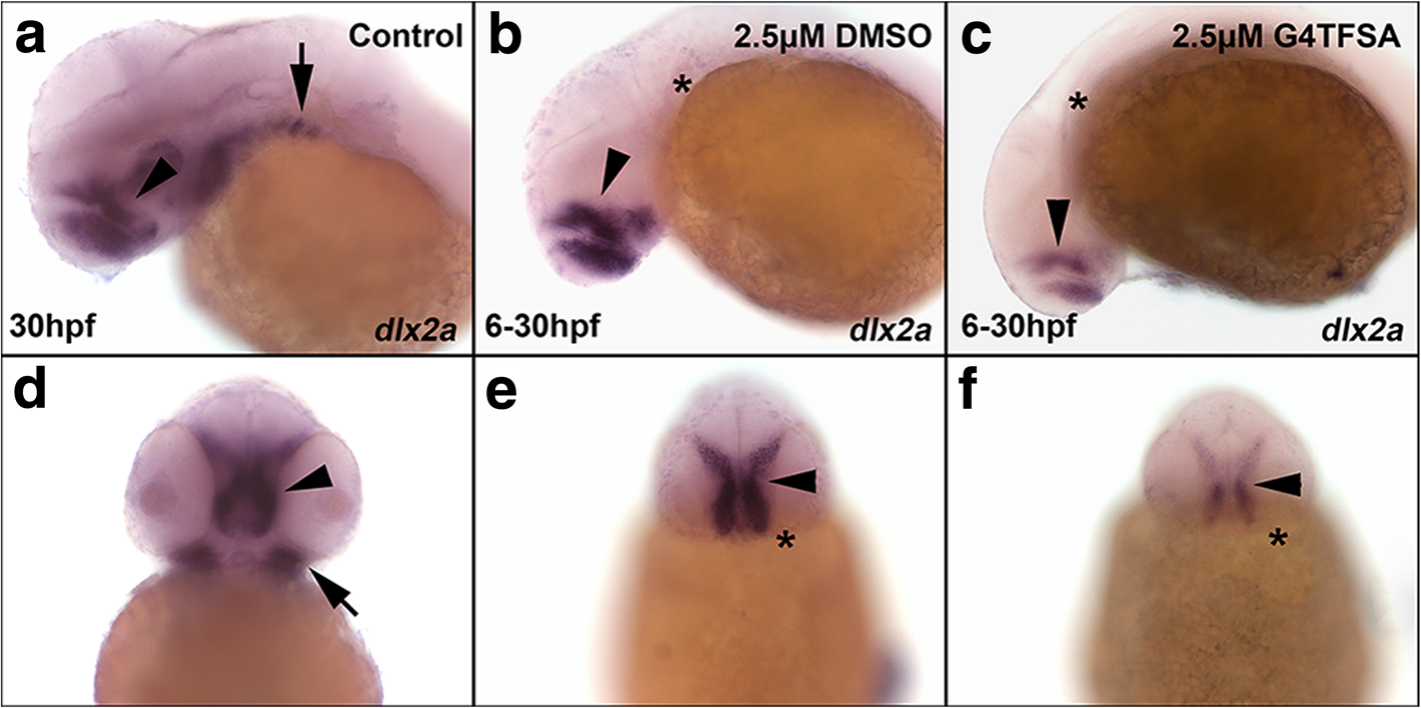
Expression of *dlx2a* visualized using whole mount *in situ* hybridization. Embryos exposed to SU5402 at 2.5 μM in either DMSO (**b** & **e**) or G4TFSA (**c** & **f**) display reduced localization and expression of *dlx2a* compared to control (DMSO exposed only (**a** & **d**) in the hindbrain (arrow) and in the pharyngeal arches (arrow head, * lack of pharyngeal arches). The first row depicts the embryos in a later orientation, the second row depicts the embryos in a ventral orientation

With these data in hand, our attention turned to demonstrating the generality of these ionic liquids as a storage and delivery media for small molecular therapeutics. For the purpose of this study, we evaluated the ability of both G3TFSA and G4TFSA to solubilise and compare the performance of SU5402 (Fig. 4d) a pan-fibroblast growth factor receptors (FGFR) specific tyrosine kinase inhibitor and is used in a multitude of zebrafish developmental systems to specifically inhibit FGFR signalling against DMSO [39–43]. A comparison of zebrafish embryos treated with both SU5402 in G4TFSA at concentrations 2.5 μM (Fig. 4c) and 5 μM (Fig. 4f) from 6 to 30 hpf displayed previously reported phenotypes, such as lack of pectoral fin induction (arrowhead) malformation of the otic vesicle (arrow) compared to the control embryo (Fig. 4a). Embryos treated with SU5402 in DMSO (Fig. 4b) also reported these phenotypes. There was also an evident lack of a formed mid-hindbrain boundary at concentrations of only 5 μM DEAB in DMSO and G4TFSA (open arrowhead) (Fig. 4e, f) [38, 44].

### Assessing penetrance of the SILs

In addition, we wanted to the examine genes that are affected by FGF (fibroblast growth factor) signalling. Hence, we investigated the effect on *dlx2a* expression using whole mount *in situ* hybridisation (WISH) after exposure to 2.5 μM SU5402 that had been either solubilized in DMSO or G4TFSA (Fig. 5). WISH results showed that compared to the control embryos (Fig. 5a, d), embryos treated with SU5402 solubilized in both DMSO (Fig. 5b, e) and G4TFSA (Fig. 5c, f) depicted an absence of *dlx2a* expression in the ventral cranial neural crest cells (arrows) marked by an asterix. However, comparatively there was a greater reduction in the expression of *dlx2a* (marked by AP staining) in the telencephalon (arrowhead) in zebrafish embryos that were treated with SU5402 solubilized in G4TFSA (Fig. 5c, f) compared to those treated with SU5402 solubilized in DMSO (Fig. 5b, e). This indicates that G4TFSA has as greater ability to deliver SU5402 (penetrative power) compared to DMSO.

## Discussion

Previous studies have shown that the prolonged storage of organic compounds in solution can lead to significant sample degradation, and subsequently an increase in the number of false positives for high-throughput biological screening assays [45]. These false positives represent potentially erroneous investment of time and money elaborating on a false lead compound. As a result, many researchers and pharmaceutical organizations now store their organic compounds as frozen DMSO stock solutions in an environment of low relative humidity, to mitigate or retard compound degradation [45]. A study conducted by Kozikowski et al., investigating the effect of room temperature storage on the stability of compounds in DMSO concluded that the relationship between length of storage and the probability of observing the compound is described by a repeated measures logistic regression model [45]. Results from the study determined that the probability of observing the compound was 92% after 3 months of storage at room temperature, 83% after 6 months, and 52% after 1 year in DMSO [45]. Hence, it is valuable to assess the long term effects of RT storage on DEAB penetrance post solubilisation in both G3TFSA and GF4TFSA.

Furthermore, a key characteristic of DMSO is that compared to G4TFSA it has a relatively higher melting point (T_m_) of around 19 °C [46], hence DMSO freezes easily and remelts slowly at room temperature. This means that if stored frozen, a considerable amount of time will be spent getting DMSO (and the compound solubilized in DMSO) to a liquid state before it can be used. Whereas, G4TFSA was able to remain in a liquid like state at − 20 °C, proving advantageous over DMSO as it can be used straight away after being removed from storage at − 20 °C. Similar to DMSO, G3TFSA albeit higher, has a similar T_m_ of 23 °C [47]. However, G3TFSA has a much higher entropy change of fusion value of 112.5 J K^− 1^ mol^− 1^ compared to DMSO which only has an entropy change of fusion value of around 50 J K^− 1^ mol^− 1^ [47]. This means that even though both G3TFSA and DMSO have similar melting temperatures, G3TFSA will return to a liquid state much quicker than DMSO. Our 10 mL stock of G3TFSA stored at − 20 °C took approximatively 10 min to melt at room temperature (around 20 °C) while it took slightly over 1 h for the 10 mL stock of DMSO stored at − 20 °C to completely melt at room temperature making G3TFSA to return to a liquid state at least 6 time faster than DMSO.

Consequently, a study looking in to the effect of freeze/thaw cycles on the stability of compounds in DMSO concluded that samples that underwent freeze/thaw cycling suffered the most, showing a drop of more than 10% in compound efficacy within 10 cycles [48], and after 25 freeze/thaw cycles tested, the percentage of compound remaining was 55.8% [48]. Hence, since G4TFSA does not freeze at -20 °C, there is no risk of compound degradation due to freeze/thaw cycles.

Moreover, SU5402 was used as a drug of choice in evaluating the efficacy of the SILs as it is useful for assessing requirements for FGF signalling in the later stage of development of the zebrafish embryo because it can be applied in late developmental events such as organogenesis, leaving early FGF-dependent processes unaffected. Additionally, SU5402 treatment potentially uncovers FGF requirements that are not revealed by knocking out specific FGF ligands or receptors owing to redundancy.

In making up the stock solution of SU5402 in the respective solvents; DMSO, G3TFSA and G4TFSA, it was observed that SU5402 was not soluble in G3TFSA. While the structure of both solvate ionic liquids is very similar, and they possess similar physical properties, the poly-ether used to fabricate G4TFSA possesses an extra ethylene unit potentially increasing the solubilising power of this liquid for small organic molecules [5]. Hence, only SU5402 solubilized in G4TFSA was used to assess SU5402 phenotypic penetrance against SU5402 solubilized in DMSO.

A previous study by Gibert et al., reported the formation of oral and pharyngeal dentition in teleosts depends on differential recruitment of retinoic acid signalling and that a lack of FGF signalling affects the expression of *dlx2a* (distal-less homeobox 2a) [49]. *Dlx* genes are expressed in a coordinate manner which create proximal to distal polarity within the pharyngeal arches [50]. In zebrafish, *dlx2a* is expressed in the migrating cranial neural which contributes to the pharyngeal arches [49–53].

In order to get a more accurate understanding into whether these SILs can be used clinically we will need to continue our studies in adult and mammalian models. Even though zebrafish provide an adequate starting point, the efficacy and penetrance of G3TFSA and G4TFSA would need to be assessed in a mammalian model which would share more homology to humans. Furthermore, by using these models, we could look into more reported effects known to be caused by these treatments.

Besides, in order to fully establish whether G3TFSA and G4TFSA can be used as replacement organic solvents, the efficacy to deliver a wide range of compounds and therapeutics needs to be assessed. The best way to do this would be to use an established drug/compound library which has an established and comprehensive account of observed side effects and phenotypes.

## Conclusions

Our data reveals that both G3TFSA and G4TFSA are comparable, at least, or slightly superior to DMSO in terms of compound deliverance as exhibited by the penetrance of DEAB and SU5402 (the latter only soluble in both DMSO and G4TFSA). This was evident by DEAB solubilized in G3TFSA and G4TFSA exhibiting the same phenotypic characteristics of DEAB made up in DMSO. SU5402 solubilized in G4TFSA and DMSO, reduced the expression of *dlx2a* in zebrafish embryos although to a greater extent for the latter. However, in regards to the storage of DEAB stock solutions in DMSO, G3TFSA and G4TFSA in the more conventional manner at − 20 °C, G4TFSA remained in a liquid state as it had a much lower glass-transition temperature, potentially realising a decreased rate of sample degradation. Consequently, G3TFSA and G4TFSA solubilize and deliver test/pharmacological compounds adequately and routinely in research laboratories, hence both G3TFSA and G4TFSA are suitable replacements for DMSO for experimental procedures.

## Methods

### Animal husbandry

Zebrafish were reared and staged at 28.5 °C according to Kimmel et al. [54] After spawning, embryos were collected and raised in a petri dish in embryonic medium E3. As a standard, we raised zebrafish embryos in 30 ml of E3 with 60 embryos per tube.

### Treatments

N,N-Diethylaminobenzaldehyde (DEAB) (Sigma, MO, USA) and SU5402 (Sigma, MO, USA) were dissolved in different solvents: DMSO, G3TFSA and G4TFSA at a concentration of 10 mM. This concentration was chosen based on our previous published data on the toxicity of these solvents [29]. Stock solutions on 10 mM were kept stored in either -20 °C or RT (only DEAB stocks) depending on the application. After vortexing, appropriate volumes were used to expose zebrafish embryos from 6 to 30 hpf in the dark. Live imaging was performed at 30 hpf.

### Whole mount *in situ* hybridisation

Embryos were fixed in 4% PFA-PBST overnight at 4 °C and then transferred to and stored in 100% methanol at -20 °C. Whole-mount *in situ* hybridization using digoxigenin-labeled riboprobes was performed as previously described [49]. Using *distal-less homeobox 2a* (*dlx2a)* as a probe, whole-mount *in situ* hybridization was performed on at least 20 embryos (10 treated embryos and 10 control embryos).

## Abbreviations

AP: Alkaline phosphatase
DEAB: N,N-diethylaminobenzaldehyde
DMSO: Dimethyl sulfoxide
dpf: Days post fertilization
G3TFSI: Triglyme
G4TFSI: Tetraglyme
hpf: Hours post fertilization
IL: Ionic liquid
LiNTf_2_: Lithium bistrifluoromethanesulfonimide
RA: Retinoic acid
RT: Room temperature
SIL: Solvate ionic liquid
T_m_: Melting temperature
WT: Wild type
ZF: Zebrafish
